# Self-Confidence, Not Self-Awareness, Is Negatively Associated With Areca Nut Dependence

**DOI:** 10.3389/fnut.2022.898264

**Published:** 2022-07-06

**Authors:** Chen-Yuan Hsu, Pei-Chi Chang, Sheng-Lei Yan

**Affiliations:** ^1^College of Nursing and Health Sciences, Dayeh University, Changhua, Taiwan; ^2^Chia-Yi Christian Hospital, Chiayi City, Taiwan; ^3^Division of Gastroenterology and Hepatology, Department of Internal Medicine, Chang Bing Show Chwan Memorial Hospital, Changhua, Taiwan; ^4^College of Biotechnology and Bio-resources, Dayeh University, Changhua, Taiwan

**Keywords:** self-confidence, self-awareness, areca nut, dependence, areca nut cessation

## Abstract

Areca nut is the fourth most commonly used addictive substance globally. Therefore, this study aimed to examine correlations among areca nut self-awareness, areca nut cessation self-confidence, and areca nut dependence in the Taiwanese population. This was a descriptive study in which 120 areca nut chewers who sought medical attention at a regional hospital and were residents of the Yunlin-Chiayi area, were recruited as study subjects. A structured questionnaire was used for data collection, which included demographic data, an areca nut self-awareness scale, an areca nut cessation self-confidence scale, and an areca nut dependence scale. A Pearson correlation analysis revealed that areca nut self-awareness and areca nut cessation self-confidence were not significantly correlated (*r* = 0.16, *p* = 0.069). Areca nut self-awareness and areca nut dependence also did not have a significant correlation (*r* = −0.06, *p* = 0.511). However, we found that areca nut cessation self-confidence and areca nut dependence were significantly negatively correlated (*r* = −0.37, *p* < 0.001), that is, the higher the areca nut cessation self-confidence, the lower the areca nut dependence. In addition, areca nut self-awareness showed significant correlations by age (*r* = 4.54, *p* = 0.005), occupation (*r* = 2.91, *p* = 0.02), and family support (*r* = 3.83, *p* = 0.03). Scheffe's *post-hoc* test revealed significant differences that subjects whose family members were extremely supportive of areca nut cessation had higher areca nut self-awareness. In conclusion, areca nut cessation self-confidence and areca nut dependence showed a significant negative correlation. Areca nut self-awareness revealed significant correlations by age, occupation, and family support. The results of this study can be used to provide a reference for implementing areca nut cessation policies in the future.

## Introduction

Areca nut is classified as group 1 carcinogen and is also the fourth most common addictive substance worldwide after coffee, cigarettes, and alcohol (1, 2). Areca nut chewing is associated with cancers, such as esophageal cancer, pancreatic cancer, laryngeal cancer, and lung cancer (3) as well as oral submucous fibrosis (OSF) (4) and oral cavity carcinomas (5). Evidence has also shown that areca nut chewing is known to cause addiction (6, 7). The habit is extremely common in Taiwan, where most areca nut chewers are aged 25–44 years, and the number of these specific nut chewers totals around 100,000 people (8). Although the Taiwanese government has strongly warned of the harm caused by areca nut chewing and greatly promoted areca nut cessation classes in recent years, their effectiveness has been limited.

Areca nut chewing may lead to substance dependence (9). Studies have shown that long-term areca nut chewing may lead to habits, dependence, and addiction (9, 10). In addition, withdrawal symptoms, such as unstable emotions, anxiety, irritability, inability to concentrate, sleep disturbances, and need for areca nut (11), would occur during areca nut cessation (3, 12–17). Therefore, it is essential to develop a referential indicator correlated to areca nut dependence, to drive areca nut cessation policies.

Self-confidence is associated with persistence in task completion, execution capabilities, focus, and positive energy, and is the most significant factor that may determine whether an individual is successful in executing certain tasks. The main self-confidence theory examined in this study is the self-efficacy theory, which was proposed by psychologist Bandura in 1976 and based on sociological theory development. This theory states that execution of health behavior is affected by personal perception, behavior, environmental factors, etc. (18–21). Therefore, self-confidence may be a good indicator to evaluate the determination for areca nut cessation.

Self-awareness is a reflective process that involves attention being inwardly focused toward the self rather than outwardly focused toward the environment (22). Evidence has shown that areca nut dependent chewing is associated with higher rates of impairment of self-awareness, inhibitory control, and decision-making (23). Therefore, understanding the state of areca nut chewers' self-awareness in terms of their perception of the hazards of areca nut may be helpful for front-line health professionals in assisting them to quit areca nut chewing.

The aim of this study was to examine the relationship between self-awareness, self-confidence, and dependence among areca nut chewers by using a questionnaire. The theory applied in this study included effects of awareness in areca nut chewers (e.g., benefits of areca nut cessation on me), behavioral effects (e.g., how do I quit areca nut chewing), and environmental effects (e.g., support from friends and family in areca nut cessation).

## Materials and Methods

### Study Design and Participants

A descriptive study with questionnaire-based survey was employed for this study. The aim was to examine the correlation among three factors: areca nut self-awareness, areca nut cessation self-confidence, and areca nut dependence in areca nut chewers.

Convenience sampling was employed for this study. People who sought medical attention at the outpatient clinic of a regional hospital, and community residents who chewed areca nuts in the Yunlin-Chiayi area were recruited as study subjects. In particular, we recruited people who underwent oral mucosa screening in the hospital and were community residents who attended areca nut control health education advocacy, estimation the number of samples was 80–100, and finally the actual number of recruited samples 120 was employed for statistical analysis. After the study protocol was explained, subjects were enrolled if they provided consent for participating; they all signed informed consent forms.

The inclusion criteria were as follows: (i) Areca nut chewers aged 20 years or above; (ii) Willing to participate in this study after the aim of the study was explained; and (iii) Able to communicate clearly. The exclusion criteria were as follows: (i) People with impaired consciousness or were unable to clearly express themselves; (ii) Those who were currently undergoing areca nut cessation; and (iii) Those who were diagnosed with oral cancer and were currently undergoing treatment.

### Study Tools

A structured questionnaire developed for this study was used for data collection. Scales assessing areca nut self-awareness, areca nut cessation self-confidence, and areca nut dependence were then administered to all participants. The questionnaire (24) included the following four sections:

(i) Demographic data: Name, gender, year of birth, educational level, occupation, smoking, alcohol consumption, oral mucosa screening status, and support for areca nut cessation from family and friends (24).
(ii) Areca nut self-awareness scale: With regard to the effects of areca nut on physical health, the subjects selected true or false answers. There are 12 questions, a total score of 12 scores. For example, “people who chew areca nut for a long time are prone to white spots on the oral mucosa”, "people who chew areca nut for a long time may have difficulty in opening their mouth again, and “difficulty opening mouth and tongue movement are signs of oral precancerous lesions”. Correct answers were scored as 1 point and wrong answers as 0. The higher score means the higher of the areca nut self-awareness. Experts were invited to assess the relevance of the content of the questions using a 5-point scale. The higher the score, the more suitable the question was for this study. The experts were composed of five professional clinical doctors, nurses, and researchers from Taiwan. The expert-rated content validity index (CVI) was 0.98 (24).
(iii) Areca nut cessation self-confidence scale: This scale contained eight questions, for example, “I see other people successfully quit areca nut, I can be like them” and “I am confident that I can quit areca nuts.” The scale used a Likert-style 4-point scale (4 = strongly agree, 3 = agree, 2 = disagree, 1 = strongly disagree) and the higher score equated to higher areca nut cessation self-confidence. The expert-rated CVI was 0.93 (24).
(iv) Areca nut dependence scale: This scale was divided into three dimensions, including “craving,” “usage habit,” and “withdrawal response,” with a total of 11 questions. For example, “I now have the desire to eat areca nuts,” “When there is no areca nut around, I will always look for areca nuts,” and “When I chew areca nuts, I usually chew several pieces.” The scale used a Likert-style 4-point scale (4 = strongly agree, 3 = agree, 2 = disagree, 1 = strongly disagree. The higher score indicated higher areca nut dependence, and a total score of 24 or more was regarded as addiction. The internal consistency reliability Cronbach' s α was 0.89. The correlation coefficients among the three-dimension subscales were between 0.44 and 0.511, which reached a significance level of 0.001. The expert-rated CVI was 0.98 (24).

### Statistical Methods

After the questionnaires were collected and compiled, the SPSS 22.0 statistical software was used for descriptive and inferential analysis of the source data. A significance level of 0.05 was used for all statistical tests. The following section presents the statistical analyses carried out on the data:

### Descriptive Statistics

The number of subjects and percentages were used to describe the gender, age, educational level, occupation, smoking, alcohol consumption, oral mucosa screening status, and support for areca nut cessation from family and friends. Continuous data were expressed as means and standard deviations, and used to describe areca nut self-awareness, areca nut cessation self-confidence, and areca nut dependence.

### Inferential Statistics

An F test was used to analyze in areca nut self-awareness, areca nut cessation self-confidence, and areca nut dependence by demographic variables. Scheffe's test was used for *post-hoc* testing in analysis of variance (ANOVA) of variables. Pearson's correlation coefficients were calculated to examine the correlation among areca nut self-awareness, areca nut cessation self-confidence, and areca nut dependence.

### Ethical Approval

This study was approved by the institutional review board of the study institution (CYCH-IRB No: 106029). All study participants provided written informed consent before the questionnaires were administered to them.

## Results

### Basic Information Analysis

A total of 120 areca nut chewers participated in the study, of which 119 were men and 1 was a woman. Subjects in the age group of 41–50 years accounted for the highest proportion (28.33%). The majority of the subjects reported an educational level of senior high school (39.17%). The most common occupations were in the category of “other” as well as in agriculture, forestry, fishing, animal husbandry, and industry. Most of the areca nut chewers were smokers (75.8%), and did not consume alcohol (55.8%). A total of 61.67% of the subjects had previously undergone oral mucosa screening. The families of 68.33% of the subjects were “extremely supportive” of areca nut cessation. Most of the participants' friends (52.5%) “did not have any particular opinion” on areca nut cessation. Table 1 shows the basic information analysis.

**Table 1:** Basic information analysis (*n* = 120).

**Variable**	**Item**	**n**	**Percentage (%)**
Gender	Male	119	99.17
	Female	1	0.83
Age	≤ 40	32	26.67
	41– 50	34	28.33
	51–60	26	21.67
	>60	28	23.33
Education level	None	2	1.66
	Elementary school	27	22.50
	Secondary school (junior high)	33	27.50
	Senior high school (vocational school)	47	39.17
	Junior college, undergraduate	9	7.50
	Graduate school and above	2	1.67
Occupation	Military, civil service, education	7	5.8
	Service industry	12	10
	Electronics and communications industry	5	4.2
	Manufacturing industry	20	16.7
	Agriculture, forestry, fishing, and animal husbandry	26	21.7
	Other	50	41.6
Smoking	Yes	91	75.8
	Quit smoking	15	12.5
	No	14	11.7
Alcohol consumption	Yes	53	44.2
	No	67	55.8
Oral mucosa screening	Never	39	32.50
	Undergone previously	74	61.67
	Once every 2 years	7	5.83
Family support	Extremely supportive	82	68.33
	No particular opinion	25	20.83
	Let it be	13	10.84
Support from friends	Extremely supportive	39	32.50
	No particular opinion	63	52.50
	Let it be	18	15.00

### Correlation Analysis Among Areca Nut Self-Awareness, Areca Nut Cessation Self-Confidence, and Areca Nut Dependence

Pearson correlation coefficients were calculated to analyze the correlations among areca nut self-awareness, areca nut cessation self-confidence, and areca nut dependence (Table 2). Although the correlation between areca nut self-awareness and areca nut cessation self-confidence was not significant (*r* = 0.16, *p* = 0.069), there was a positive trend. The correlation between areca nut self-awareness and areca nut dependence was also not significant (*r* = −0.06, *p* = 0.511), but showed a negative trend. Areca nut cessation self-confidence and areca nut dependence showed a statistically significant negative correlation (*r* = −0.37, *p* < 0.001). From this, we can see that the higher the areca nut cessation self-confidence, the lower the areca nut dependence. In contrast, subjects with high areca nut dependence lacked self-confidence for areca nut cessation.

**Table 2:** Correlation analysis among areca nut self-awareness, areca nut cessation self-confidence, and areca nut dependence (*n* = 120).

**(M±SD)**	**Areca nut self-awareness**	**Areca nut cessation self-confidence**	**Areca nut dependence**
Areca nut self-awareness (8.02 ± 2.5)	1	0.16	−0.06
Areca nut cessation self-confidence (20.74 ± 2.85)		1	−0.37^***^
Areca nut dependence (26.72 ± 5.52)			1

*** *p < 0.001*.

### Analysis of Correlation in Various Demographic Variables by Areca Nut Self-Awareness, Areca Nut Cessation Self-Confidence, and Areca Nut Dependence

The results of the analysis of correlation in areca nut self-awareness, areca nut cessation self-confidence, and areca nut dependence among various demographic variables are illustrated in Table 3. We found that only areca nut self-awareness showed significant correlation by age (*r* = 4.54, *p* = 0.005), occupation (*r* = 2.91, *p* = 0.02), and family support (*r* = 3.83, *p* = 0.03). The other variables did not show significant correlation with any of the three factors, that is, areca nut self-awareness, dependence, or cessation self-confidence.

**Table 3:** Analysis of correlation in areca nut self-awareness, areca nut cessation self-confidence, and areca nut dependence by various demographic variables (*n* = 120).

**Variable**	**Areca nut self-awareness**	**Areca nut cessation self-confidence**	**Areca nut dependence**
	***F*-value**	***F*-value**	***F*-value**
Age	4.54^**^	1.58	1.38
Education level	1.58	1.10	0.58
Occupation	2.91^*^	1.62	2.18
Smoking	0.23	2.76	1.69
Alcohol consumption	1.77	−0.56	−0.62
Oral mucosa screening	0.96	1.53	0.23
Family support	3.83^*^ (1 > 3)^a^	2.02	0.96
Support from friends	1.64	0.10	1.69

a *Scheffe's post-hoc analysis was conducted as follows: a family support variable of 1 represents extremely supportive, 2 represents no particular opinion, and 3 represents let it be*.

* *Significant at p < 0.05*.

** *Significant at p < 0.01*.

Further, Scheffe's test *post-hoc* analysis showed that there were differences in areca nut self-awareness by family support; a family support variable of 1, 2 and 3 represented extremely supportive, no particular opinion and let it be (e.g., go with the flow), respectively. The total areca nut self-awareness score was higher for subjects who rated family support as “extremely supportive” (8.38 ± 2.18), compared to those who described their family's attitude as “let it be” (6.46 ± 3.36). There were no significantly differences in areca nut self-awareness by age and occupation variables of Scheffe's test (Table 3).

## Discussion

This study examined the correlations among areca nut self-awareness, areca nut dependence, and areca nut cessation self-confidence. It also examined the correlations in areca nut self-awareness, dependence, and cessation self-confidence based on various demographic variables. A previous study reported that areca nut self-awareness affects areca refusal self-efficacy and that increased awareness aids areca nut cessation (25). However, this study found that there is no significant correlation between areca nut cessation self-confidence and areca nut self-awareness, that is, the ratio of correct answers on areca nut-related knowledge is not correlated with areca nut cessation self-confidence. This result may be largely related to the study population, as previous studies did not survey areca nut chewers alone, but compared self-awareness results between areca nut chewers and non-areca nut chewers or ex-chewers. In contrast, our study surveyed only areca nut chewers and employed a cross-sectional study design in which only the responses from current interviewees were used for analysis. Therefore, the scores of areca nut self-awareness did not differ greatly, and the standard deviation was only 2.5 points. This shows that the subjects had comparable self-awareness and it was difficult to find a significant correlation in areca nut cessation self-confidence by level of self-awareness, among them.

Although knowledge can improve self-efficacy, Bandura (19, 26) stated that if an individual believes that a certain behavior can produce definite outcomes; this does not mean that he/she has the self-confidence to carry out this behavior. To date, there is no relevant literature examining the correlation between areca nut self-awareness and areca nut dependence. The results of this study only reveal that areca nut self-awareness and dependence show a negative trend. From the results of areca nut self-awareness, we found that only 60% of subjects answered correctly for “areca nut chewing can lead to addiction.” However, more than 70% of the subjects in this study had an addiction diagnostic by the areca nut dependence scale. This shows that many subjects were unaware of their own addiction. This result may indicate a potential correlation between areca nut self-awareness and dependence.

Although areca nut self-awareness had no significant correlation with areca nut cessation self-confidence and areca nut dependence. We can see that the mean percentage of correct answers for areca nut self-awareness was 70.41%, and more than 80% of the subjects answered correctly for the self-awareness about precancerous oral lesions, but only 50% answered correctly for chronic diseases caused by areca nut chewing. This shows that the Taiwanese government's oral cancer prevention advocacy has achieved some results in recent years. However, education and self-awareness of the harm caused by areca nut chewing on physical health is still not widespread and needs to be expanded.

This study found that areca nut cessation self-confidence and areca nut dependence showed a significant negative correlation. This means that areca nut dependence is lower when areca nut cessation self-confidence is higher. From this, it can be concluded that strengthening areca nut cessation self-confidence, such as it is perhaps more likely that lower dependence leads to higher self-confidence, may lead to successful areca nut cessation results. Studies on areca nut abusers also found that high refusal self-efficacy reduced future areca nut chewing behavior (27), which is consistent with the results of this study.

This study showed that men account for the majority of the areca nut chewing population in Taiwan at present, and most of them have never undergone oral mucosa screening. However, most family members are extremely supportive of areca nut cessation. This shows that the best method for encouraging areca nut cessation is to use “family expectations” and “to think about the family” to influence people's willingness to quit areca nut chewing. This result is consistent with a previous study (28), which highlighted an important influence of strengthened family support. In addition, the self-awareness of oral mucosa screening can be improved in these families to encourage areca nut chewers to undergo screening.

This study showed that areca nut chewers under the age of 40 accounts for 26.67%, it can be seen from this; areca nut chewers form the habit of chewing areca nuts in adulthood. Therefore, the prevention of areca nut chewing behavior should start with the development of relevant health promotion programs. Such educational programs should be implemented before adulthood, or even earlier during school-going age. This result is consistent with previous studies, as influence as age resulted with health promotion programs (29–31).

Our study found that areca nut cessation self-confidence and areca nut dependence showed a significant negative correlation. Therefore, self-confidence is an important factor for ameliorating areca nut dependence. In addition, areca nut self-awareness revealed significant correlations by age, occupation, and family support. Further, family support for areca nut cessation was significant for areca nut self-awareness, suggesting that family support for areca nut cessation will affect self-awareness of areca nut-related knowledge in areca nut chewers. However, our study did not examine whether an extremely supportive family and good self-awareness are correlated with areca nut cessation effectiveness. We recommend that an in-depth study on this topic be carried out in the future.

## Limitations and Future Directions

Due to workplace and time limitations, and the fact that recruited subjects were areca nut chewers who visited a particular hospital and were part of the local community, the results cannot be generalized to people living in other regions. As well, the sample only included one woman and therefore is only representative of men. Another limitation is the small sample size. We recommend working with hospitals in different counties and cities within Taiwan in the future, which may reveal the patterns of regional differences. In addition, a cross-sectional study design was used to collect data for this study, with a focus on the relationship among different variables. Therefore, we are unable to deduce causality in the present study.

## Data Availability Statement

The original contributions presented in the study are included in the article/supplementary material, further inquiries can be directed to the corresponding author/s.

## Ethics Statement

The studies involving human participants were reviewed and approved by Chia-Yi Christian Hospital, the Institutional Review Board of CYCH-IRB No: 106029. The patients/participants provided their written informed consent to participate in this study.

## Author Contributions

C-YH: conceptualization, formal analysis, resources, and writing original draft preparation, methodology, software, data curation, writing—review and editing, visualization, supervision, project administration, and funding acquisition. C-YH, P-CC, and S-LY: validation. P-CC: investigation. All authors contributed to the article and approved the submitted version.

## Funding

The APC was funded by C-YH.

## Conflict of Interest

The authors declare that the research was conducted in the absence of any commercial or financial relationships that could be construed as a potential conflict of interest.

## Publisher's Note

All claims expressed in this article are solely those of the authors and do not necessarily represent those of their affiliated organizations, or those of the publisher, the editors and the reviewers. Any product that may be evaluated in this article, or claim that may be made by its manufacturer, is not guaranteed or endorsed by the publisher.
